# Diagnostic Approaches to Helicobacter pylori: A Comparative Study of Detection in Gastric Biopsy and Aspirates

**DOI:** 10.7759/cureus.57100

**Published:** 2024-03-28

**Authors:** Radha Kumari, Manoj Kumar, Kumari Seema, Abhay Kumar, Manju Boipai, Manohar Lal Prasad, Ashok K Sharma

**Affiliations:** 1 Microbiology, Rajendra Institute of Medical Sciences (RIMS), Ranchi, IND; 2 Medicine, Rajendra Institute of Medical Sciences (RIMS), Ranchi, IND

**Keywords:** endoscopic-guided biopsy, gastric aspirate, pcr, rapid urease test, helicobacter pylori

## Abstract

Background

*Helicobacter pylori* is one of the most common bacterial pathogens in humans. It is a microaerophilic bacteria with multiple unipolar flagella. It is associated with the development of various lesions like chronic gastritis, gastric ulcers, adenocarcinoma, and mucosa-associated lymphomas. The aim of this study was a comparative evaluation of the rapid urease test (RUT) and polymerase chain reaction (PCR) in gastric biopsy and aspirates for the detection of *H. pylori *infection and to further determine the sensitivity and specificity of RUT and PCR.

Method

Endoscopic guided biopsy tissue and gastric aspirate specimens were collected from 110 patients with symptoms like gastritis, dyspepsia, etc., and subjected to RUT and PCR for detection of *H. pylori* infection.

Results

A total of 110 samples, including both biopsy tissue (77) and gastric aspirate (33) were subjected to RUT and PCR. RUT for biopsy tissue showed the highest sensitivity (97.18%), compared to gastric aspirate (78.94%). Comparing RUT with PCR, the sensitivity and specificity of PCR were 93.33% and 90.0%, respectively. The positive predictive value (PPV) of PCR was 97.67%, the negative predictive value (NPV) was 75.0%, and the accuracy was 92.73%.

Conclusion

The present study showed that RUT is a rapid and accurate invasive test for the detection of *Helicobacter pylori* infection in biopsy tissue as compared to gastric aspirate specimens, which are more sensitive to PCR. The study also showed that biopsy tissue was found to be a superior specimen for the detection of *Helicobacter pylori *as compared to gastric aspirate.

## Introduction

*Helicobacter pylori* is a spiral-shaped, gram-negative bacteria that colonizes the stomachs of 50% of the world's population. It is associated with peptic ulcer disease and gastric carcinoma. It is a microaerophilic bacteria with multiple unipolar flagella that was identified in 1982 by Marshall and Warren [1,2]. In 1983, scientists Warren and Marshall discovered *H. pylori* in mucosal specimens of patients with chronic active gastric and peptic ulcers [1]. Humans are the only known reservoir host of *H. pylori*. The bacterium colonizes the human gastric mucosa, and human contact remains the major mode of transmission [3]. Infection with *H. pylori* manifests as chronic gastric inflammation, gastritis, and peptic ulcer disease that can sometimes result in stomach cancers like adenocarcinoma, lymphoma of the stomach, or gastric mucosal-associated lymphoid tissues (MALT) [4,5]. Approximately 4.4 billion people reportedly had an *H. pylori* infection in 2019. It is estimated that more than 50% of the world's population harbors this organism [6].

The prevalence of *H. pylori* infection is higher in developing countries like India, ranging from 49.94% to 83.30%, whereas in developed countries it is about 25 to 50% [7,8]. The symptom depends on the interaction of bacterial factors, host factors (cytokine genes or genes coding Toll-like receptors, which are at high risk of gastric adenocarcinoma), and strains of *H. pylori*. Environmental factors like smoking, high-salt diets, and preserved foods increase the risk of gastric ulcers and cancer in *H. pylori *colonized individuals. Asymptomatic infection is seen in cases colonized by less virulent strains of *H. pylori* [9,10]. Colonization of the gastric mucosa is facilitated by the spiral shape, high motility, and production of the urease enzyme, which are the important virulence factors of *H. pylori* [10]. Two proteins secreted by the bacterium that play a pivotal role in pathogenesis are cytotoxin-associated antigen (Cag A) and vacuolating cytotoxin (Vac A) [11]. The detection of Cag A and Vac A genes correlates with established *H. pylori* infection [12]. Various non-invasive and invasive methods are available for the detection of *H. pylori* infection. The non-invasive methods like H&E staining, urea breath test, and serology have low sensitivity, whereas the invasive tests consisting of rapid urease test (RUT), histopathological examination, polymerase chain reaction (PCR) performed on tissues obtained from endoscopic-guided biopsies from gastric mucosa (antrum, corpus, and body), and gastric aspirate samples are more sensitive and confirmatory [13].

As the RUT is rapid, inexpensive, and highly specific, it is the diagnostic method of choice. In 1998, the American College of Gastroenterology recommended that the RUT be the first test to be performed when endoscopy is advised [14]. *H. pylori* hydrolyzes urea to produce ammonia and carbon dioxide, and this reaction is detected by RUTs. The ammonia produced increases the pH of the RUT kit medium, resulting in a change of color to pink or red, which is observed by the naked eye. PCR accurately detects gene targets of *H. pylori* DNA, such as urease C genes, Cag A, and Hsp60, which makes it the most promising diagnostic modality. PCR can detect even a minuscule amount of bacterial DNA, but it is still considered after RUT as it requires a biopsy specimen. The accuracy of PCR depends on the choice of primers and target DNA, specimen preparations, bacterial density, and technique performed, which fluctuates according to the laboratory set-up. Our study aims to analyze and compare the diagnostic performance of RUT and PCR in gastric biopsy and aspirates for the detection of *Helicobacter pylori* infection.

## Materials and methods

The present observational prospective study was carried out in the Department of Microbiology, Rajendra Institute of Medical Sciences (RIMS), Ranchi, from April 2021 to October 2022. The relevant case history was recorded, and a detailed clinical examination was carried out. This study included endoscopic gastric mucosal biopsy specimens and endoscopic guided gastric aspirates. About 1 to 2 ml of upper gastrointestinal endoscopic gastric aspirates and two endoscopic guided biopsy samples were taken from each patient and sent to the laboratory for urease testing and polymerase chain reaction (PCR) analysis. The endoscopic biopsy sample was taken from the lesser curvature of the mid-antrum and corpus of the stomach, as in some patients, an infection may only be present in the body or antrum.

Inclusion criteria

Patients suspected of having various clinical manifestations like acute gastritis, peptic ulcer disease, chronic atrophic gastritis, autoimmune gastritis, pernicious anemia, adenocarcinoma of the stomach, gastric mucosa-associated lymphoid tissue (MALT) lymphoma, and patients aged between 18 and 60 years were included in this study. Patients taking proton pump inhibitors were also included in this study after the stoppage of drugs for two weeks.

Exclusion criteria

Critically ill patients not fit for endoscopy, pregnant women, patients with upper gastrointestinal hemorrhage, and patients on antimicrobials were excluded from the study.

Sample size

The sample size was calculated by using the sensitivity formula.

 $TP + FN = Z^2 \left [ SN\left ( 1-SN \right ) \right ] \div P^2$

$N(SN) = TP + FN / P$

Where, P = prevalence (80% from previous studies); N = sample size; TP = true positive; FN = false negative; SN = sensitivity (93%); Z = confidence interval normal distribution valve for 95%, Z= 1.96; W = accuracy = 0.05

Based on the above sensitivity formula, the sample size was calculated as 110. 

Rapid urease test

Principle 

The rapid urease test is based on the urease enzyme released by *Helicobacter pylori*, which splits the urea into ammonia and carbon dioxide. The produced ammonia increases the pH of the RUT kit medium, resulting in a color change of the RUT medium to pink or red, which is detected by the naked eye.

Test Procedure

**Figure 1:**
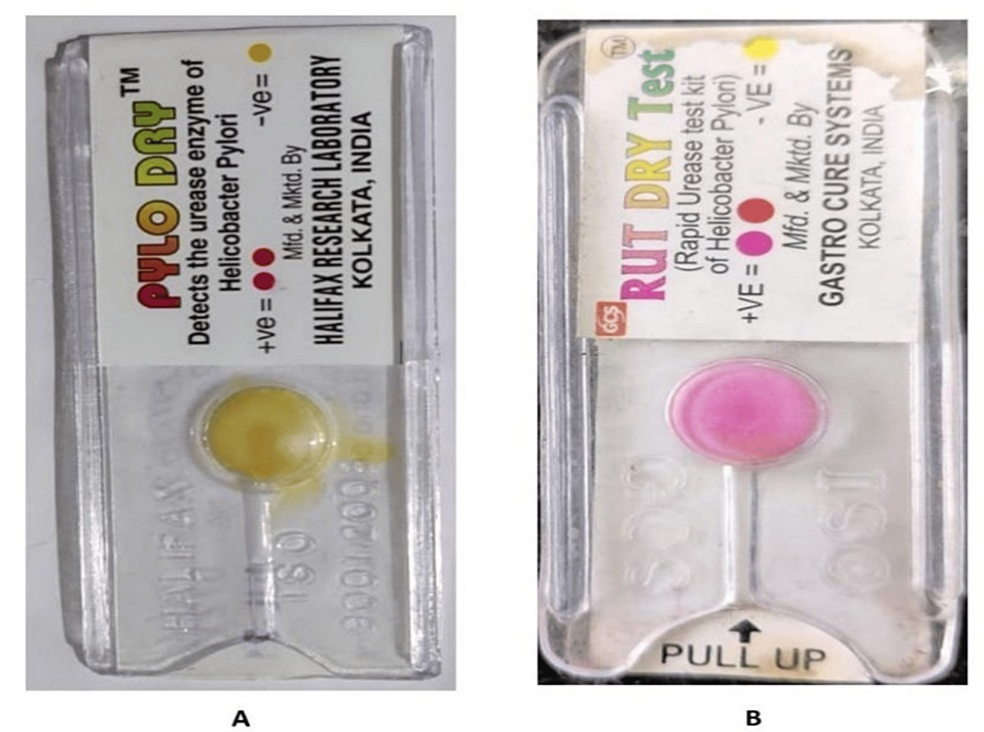
RUT kit A: negative result; B: positive result RUT: rapid urease test

A gastric aspirate sample (1 to 2 ml) and endoscopy-guided biopsy tissues were added to the well of the RUT kit (Gastro Cure Systems, Kolkata, India). A positive test for *Helicobacter pylori* was indicated by a change in the color of the medium from yellow to pink or red (Figure 1). After inoculation of the sample, if the color of the kit changed within 15 minutes, it was considered strongly positive. Whereas color change occurred within 24 hours, the test was read as weakly positive.

Endoscopic guided two gastric biopsy samples from each patient were collected according to the “Management of *Helicobacter pylori* infection: The Bhubaneswar Consensus Report of the Indian Society of Gastroenterology 2021” guideline [15]. For the RUT, at least two biopsy samples (≤25 µm) were taken, one from the corpus (middle body) and another from the lesser curvature of the mid-antrum of the stomach.

Polymerase chain reaction

The specimens were subjected to DNA extraction and PCR assays for the detection of *H. pylori* DNA. Following DNA extraction using QIAamp DNA Mini KIT (Qiagen, Germany), PCR was performed with two different primer sets, one for the Cag A gene (750 bp) and the urease C gene (375 bp). The following primers were used [16]:

Urea C-Forward [CTAGTGGTGGTGGACAATTTAGG]; Urea C-Reverse [CTTGCTTACTTTCTAACACTAACGC]; Cag750-Forward [ACAATGACTAACGAAACTATTGA]; Cag 750-Reverse [ACATCACGCCATCATGTTTTA]

The PCR reaction (SuperMix, Invitrogen) was carried out by extracting 3 µl DNA used as a template in a final volume of 25 µl containing 2 mM MgCl2, 0.2 mM of each dNTP, 0.5 µl of each primer (forward, reverse), and 1 µl of Taq DNA polymerase at an initial denaturation at 94º C for five minutes, and then denaturation at 94º C for 30 seconds, annealing temperature 50°C (at primer specific Tm) for 30 sec, followed by extension at 72º C for 30 sec of 35 cycles, and final extension at 72º C for five minutes. The amplified products were visualized on a 1.5% agarose gel with Cresol-Red dye.

Statistical analysis

The results were recorded and analyzed by MedCalc, version 20.218. Statistical analysis with the formation of a 2×2 cross table was done between gastric aspirates and biopsy tissue. The chi-square test/Fisher exact test has been used to find a significant association between findings. Diagnostic statistics like sensitivity, specificity, positive predictive value (PPV), negative predictive value (NPV), and diagnostic accuracy were obtained. P<0.05 was taken as statistically significant.

## Results

The present study consisted of a total of 110 patients with ages ranging from 18 to 60 years. In this study, 69 (62.7%) patients were male, and 41 (37.3%) patients were female. The propensity of infection increased with increasing age, with 12 (10.9%) in the age group of 20-30 years, 18 (16.4%) in the age group of 30-40 years, 35 (31.8%) in the age group of 40-50 years, and 36 (32.7%) in the age group of 50-60 years. The prevalence of *H. pylori* infection in gastritis patients was found to be 81.80%. The maximum number of patients came from rural backgrounds with lower socioeconomic status. 

Varied clinical presentations were observed in the study, with abdominal pain (88, 80%), retrosternal burning (87, 79.1%), postprandial fullness (76, 69.1%), bloating (72, 65.5%), and nausea (52, 47.3%). It was also found that diabetes mellitus type Ⅱ, hypertension, and smoking were associated with 58 (52.7%), 59 (53.6%), and 76 (69.1%), respectively. Among all the enrolled patients, 72 (65.5%) were non-vegetarian, 38 (34.5%) were vegetarian, and 78 (70.9%) were on certain medications. The correlation coefficient of dietary status with RUT results was 0.2180 (p-value = 0.0222). Out of a total of 110 samples, 77 (70%) were biopsy tissue and 33 (30%) were gastric aspirate samples. The results of biopsy tissue and gastric aspirate samples are shown in Table 1. The sensitivity and specificity of PCR as compared to RUT were 93.33% (95% confidence interval (CI) 86.05% to 97.51%) and 90.00% (95% CI 86.05% to 97.51%), as derived from contingency (2×2) (Table 2). PPV and NPV were 97.67% (95% CI 91.85% to 99.72%) and 75.00% (95% CI 53.32% to 90.24%), respectively, and accuracy was 92.73%. The correlation coefficient (r) and 95% CI for RUT were -0.4707, 0.6047 to -0.3109 (p<0.001). For PCR, the correlation coefficient and 95% CI were -0.4115, -0.5559 to -0.2429 (p<0.001). Our study found that the association of *H. pylori* infection with patients living in rural areas, nonvegetarians, histories of smoking, and ulcers was more frequent and statistically significant (Table 3).

**Table 1:** Comparison of biopsy tissue and gastric aspirates in PCR and RUT PCR: polymerase chain reaction; RUT: rapid urease test

Results of RUT & PCR	RUT Positive	RUT Negative	PCR Positive	PCR Negative
Biopsy tissue (BT) (N=77)	71	6	69	8
Gastric aspirates (GA) (N=33)	17	16	17	16
Total	88	22	86	24
Total sample: 110			Biopsy tissue: 77, Gastric aspirate: 33	

**Table 2:** Contingency table (2×2) between RUT and PCR of total samples PCR: polymerase chain reaction; RUT: rapid urease test

Result of RUT	Result of PCR		Total
	Positive	Negative	
Positive	84	2	86
Negative	6	18	24
Total	90	20	110

**Table 3:** Paired sample t-test with different comorbidities

Result of PCR	History of smoking	History of ulcer	History of dietary status	History of lower socioeconomic status with rural background
95% CI	-0.3356 to -0.5786	0.3245 to 0.1971	-0.2673 to 0.0049	-0.7769 to -0.4362
p-value	<0.0063	<0.0071	0.0587	<0.0001
Test statistic t	-2.834	2.789	-1.927	-7.121
Degree of freedom	10	5	9	6
Standard deviation	0.5422	0.3214	0.5315	0.6653
Standard error	0.0694	0.0411	0.0680	0.0851

Endoscopic findings

**Figure 2:**
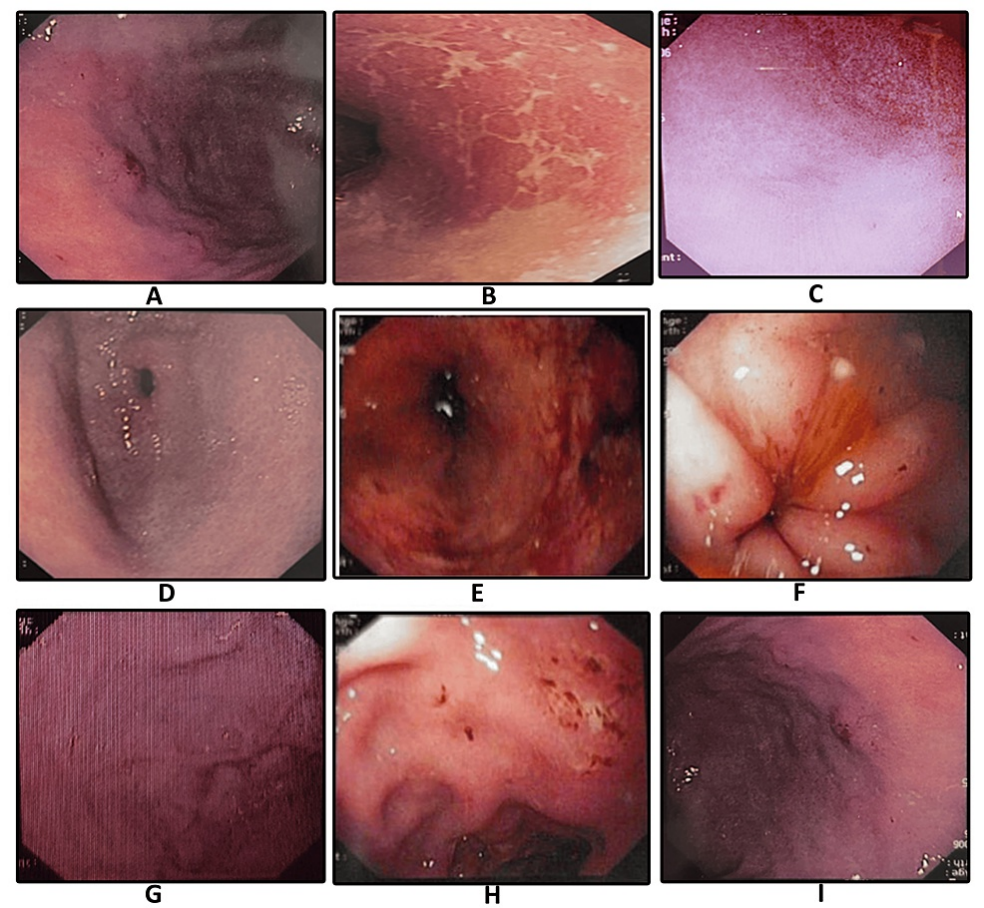
Endoscopic finding in gastric mucosa with Helicobacter pylori infection A: mucosal erosion; B: sticky mucus; C: diffuse redness; D: antral gastritis; E: spotty redness; F: duodenal ulcer; G: nodularity; H: gastric ulcer; I: enlarged fold

**Figure 3:**
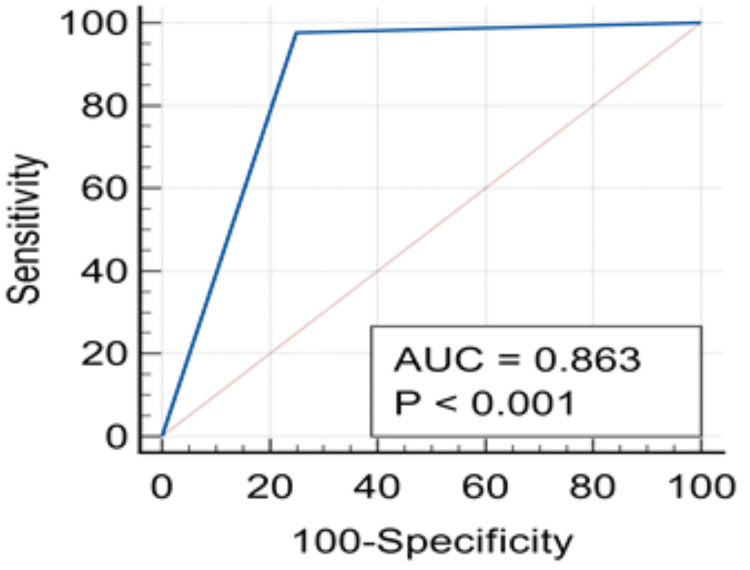
ROC curve between result of RUT and PCR PCR: polymerase chain reaction; RUT: rapid urease test; ROC: receiver operating characteristic

**Figure 4:**
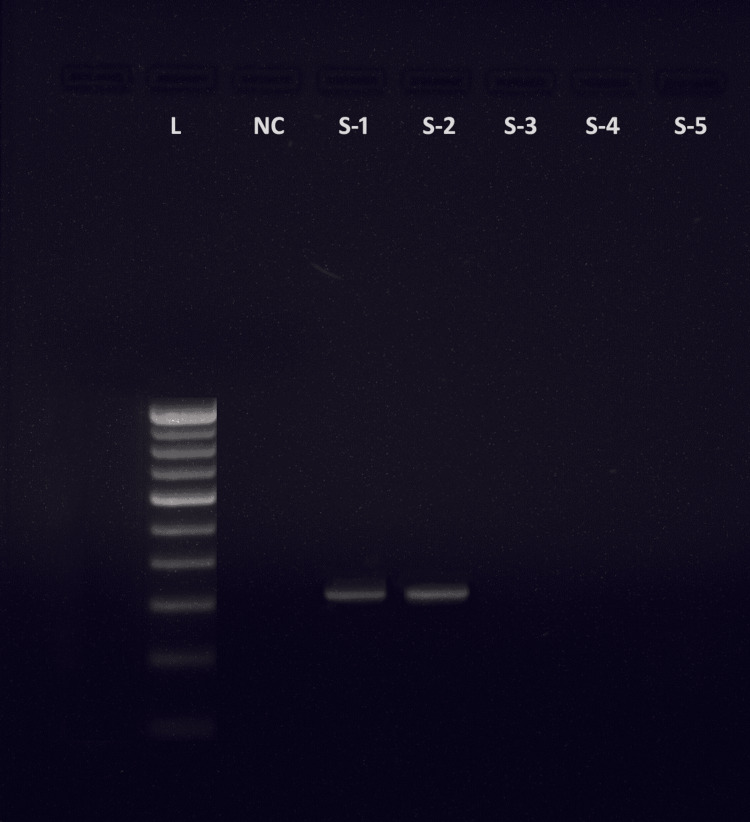
PCR gel electrophoresis using urease C gene (375 bp) Visible bands: sample no 1and 2 (positive); L: ladder; NC: negative control

Multiple superficial ulcerations (MSU) were seen at the body and antrum of the stomach as the most common endoscopic findings noted in about 52 (47.3%), followed by a mosaic pattern of mucosa (MPM) in 25 (22.7%), a duodenal ulcer at the D1 bulb (DUD1) in 15 (13.6%), multiple erosion (ME) at the antrum and fundus in 11 (10%), and the presence of nodular mucosal surface (NMS) at the body of the stomach in seven (6.4%) patients, as shown in Figure 2. The concordance or area under a curve in the ROC curve (Figure 3) was 86.3%, which was statistically significant (p<0.01). The urease C gene (375 bp) was detected by PCR (Figure 4), while the Cag A gene (750 bp) was not detected by PCR in all the samples.

## Discussion

Epidemiological studies suggest that *H. pylori* infection occurs worldwide, with varying prevalence among countries and population groups in the same country (M. Zamani et. al. 2018) [17]. It is more common in developing countries and is strongly correlated with low socioeconomic conditions. From the present study, it is evident that *H. pylori* is the causative agent for gastritis in about 81.8% of patients presenting with dyspepsia, acute/chronic gastritis, or gastric/duodenal ulcers. *H. pylori *infection is more common in males, and its prevalence tends to increase with age. *H. pylori* causes gastritis and peptic ulcers, gastric cancer, and lymphoproliferative gastric diseases in approximately 18.2% of patients.

Despite being a treatable condition, *H. pylori* infection frequently goes undiagnosed. Its early and accurate diagnosis aids in the treatment, which helps in avoiding complications. This study showed that, out of 110 patients, 78.2% were positive by RUT and 81.81% by PCR assay. The present study supported that in clinical practice if upper gastrointestinal endoscopy is indicated, RUT should be the first-line diagnostic test for detecting *H. pylori* infection as compared to PCR.

In this study, the sensitivity of RUT for biopsy tissue was 97.18%, which is consistent with the studies of Khalifehgholi M et al., Islam et al., Redéen S et al., and Cosgun Y et al. (95.6%, 93.75%, 90%, and 87.9%, respectively) [18-21]. The specificity was 83.3%, which varies and correlates with other studies like 100%, 92.86%, 98%, and 70%, respectively [18-21].

The sensitivity of RUT for biopsy tissue is often higher than that of the PCR test due to the presence of polymerase enzyme inhibitors, which negatively impact the outcome of the test. Apart from that, the PCR cannot differentiate between a live and dead organism, and it might give false positive results.

Our study also demonstrated that PCR assays can not only diagnose active infection but can also detect the urease C gene in dormant infection when the number of bacteria in the gastric mucosa is very small and may go undetected by other diagnostic methods. PCR could also constitute a very useful diagnostic tool for treatment and follow-up.

The major limitation of this study was the smaller sample size, as in the case of gastric aspirates, only 33 samples were used. Clarithromycin resistance and the presence of CYP2C19 enzymes were not detected in this study.

## Conclusions

To conclude, our study revealed that biopsy tissue samples were found to be more reliable as compared to gastric aspirates for the detection of *H. pylori* infections. RUT was a more sensitive test for biopsy tissue samples but more specific for gastric aspirate samples as compared to PCR. In the present study, we found that the PCR assay had better specificity compared to RUT. Moreover, considering the socioeconomic conditions considered during our study, the significance of these findings extends to the societal context. The preference for biopsy tissue samples could have practical implications, potentially reducing the economic burden on individuals or healthcare systems by optimizing diagnostic procedures. This insight into the comparative performance of diagnostic methods contributes to the ongoing efforts to enhance the efficiency and cost-effectiveness of *H. pylori* detection, ultimately benefiting both healthcare practitioners and individuals within diverse socioeconomic settings.

## References

[1] Ahmed N (2005). 23 years of the discovery of Helicobacter pylori: is the debate over?. Ann Clin Microbiol Antimicrob.

[2] Ishaq S, Nunn L (2015). Helicobacter pylori and gastric cancer: a state-of-the-art review. Gastroenterol Hepatol Bed Bench.

[3] Logan RP, Walker MM (2001). ABC of the upper gastrointestinal tract: epidemiology and diagnosis of Helicobacter pylori infection. BMJ.

[4] Thung I, Aramin H, Vavinskaya V, Gupta S, Park JY, Crowe SE, Valasek MA (2016). Review article: the global emergence of Helicobacter pylori antibiotic resistance. Aliment Pharmacol Ther.

[5] Correa P, Houghton J (2007). Carcinogenesis of Helicobacter pylori. Gastroenterology.

[6] Conteduca V, Sansonno D, Lauletta G, Russi S, Ingravallo G, Dammacco F (2013). H. pylori infection and gastric cancer: state of the art (review). Int J Oncol.

[7] Goddard AF, Logan RP (2003). Diagnostic methods for Helicobacter pylori detection and eradication. Br J Clin Pharmacol.

[8] van Doorn LJ, Henskens Y, Nouhan N (2000). The efficacy of laboratory diagnosis of Helicobacter pylori infections in gastric biopsy specimens is related to bacterial density and VacA, CagA, and IceA genotypes. J Clin Microbiol.

[9] Dubois A (1995). Spiral bacteria in the human stomach: the gastric helicobacters. Emerg Infect Dis.

[10] Yamaoka Y (2010). Mechanisms of disease: Helicobacter pylori virulence factors. Nat Rev Gastroenterol Hepatol.

[11] Jones KR, Whitmire JM, Merrell DS (2010). A tale of two toxins: Helicobacter pylori CagA and VacA modulate host pathways that impact disease. Front Microbiol.

[12] Andreson H, Lõivukene K, Sillakivi T, Maaroos HI, Ustav M, Peetsalu A, Mikelsaar M (2002). Association of CagA and VacA genotypes of Helicobacter pylori with gastric diseases in Estonia. J Clin Microbiol.

[13] Garza-González E, Perez-Perez GI, Maldonado-Garza HJ, Bosques-Padilla FJ (2014). A review of Helicobacter pylori diagnosis, treatment, and methods to detect eradication. World J Gastroenterol.

[14] Howden CW, Hunt RH (1998). Guidelines for the management of Helicobacter pylori infection. Ad Hoc Committee on Practice Parameters of the American College of Gastroenterology. Am J Gastroenterol.

[15] Singh SP, Ahuja V, Ghoshal UC (2021). Management of Helicobacter pylori infection: The Bhubaneswar Consensus Report of the Indian Society of Gastroenterology. Indian J Gastroenterol.

[16] Jalalypour F, Farajnia S, Somi MH, Hojabri Z, Yousefzadeh R, Saeedi N (2016). Comparative evaluation of rut, PCR and ELISA tests for detection of infection with Cytotoxigenic H. pylori. Adv Pharm Bull.

[17] Zamani M, Ebrahimtabar F, Zamani V, Miller WH, Alizadeh-Navaei R, Shokri-Shivrani J, Derakshan MH (2018). Systematic review with meta-analysis: the worldwide prevalence of Helicobacter pylori infection. Aliment Pharmacol Ther.

[18] Khalifehgholi M, Shamsipour F, Ajhdarkosh H (2013). Comparison of five diagnostic methods for Helicobacter pylori. Iran J Microbiol.

[19] Islam MDU, Shamsuzzaman SM, Muazzam N (2010). A comparative study among different invasive methods for the diagnosis of Helicobacter pylori. Faridpur Medical College Journal.

[20] Redéen S, Petersson F, Törnkrantz E, Levander H, Mårdh E, Borch K (2011). Reliability of Diagnostic Tests for Helicobacter pylori Infection. Gastroenterol Res Pract.

[21] Cosgun Y, Yildirim A, Yucel M (2016). Evaluation of Invasive and Noninvasive Methods for the Diagnosis of Helicobacter Pylori Infection. Asian Pac J Cancer Prev.

